# Supplementary material for: Effect of the Presence of Lignin from Woodflour on the Compostability of PHA-Based Biocomposites: Disintegration, Biodegradation and Microbial Dynamics
Source: Polymers (Basel). 2023 May 27;15(11):2481. doi: 10.3390/polym15112481 (PMC10255095; doi:10.3390/polym15112481)
Supplement: Supplementary file 1 [file polymers-15-02481-s001.zip › polymers-2306208-supplementary.pdf]

polymers

MDPI

Supplementary Material

Effect of the Presence of Lignin from Woodflour on the Compostability of PHA-Based Biocomposites: Disintegration, Biodegradation and Microbial Dynamics

Patricia Feijoo [1], Anna Marín [1], Kerly Samaniego-Aguilar [1], Estefanía Sánchez-Safont [1], José M. Lagarón [2], José Gámez-Pérez [1] and Luis Cabedo [1,*]

[1] Polymers and Advanced Materials Group (PIMA), Universitat Jaume I (UJI), Avenida de Vicent Sos Baynat s/n, 12071 Castelló de la Plana, Spain
[2] Novel Materials and Nanotechnology Group, Institute of Agrochemistry and Food Technology (IATA), Spanish National Research Council (CSIC), Calle Catedrático Agustín Escardino Benlloch 7, 46980 Paterna, Spain
* Correspondence: author: Luis Cabedo. Email: lcabedo@uji.es

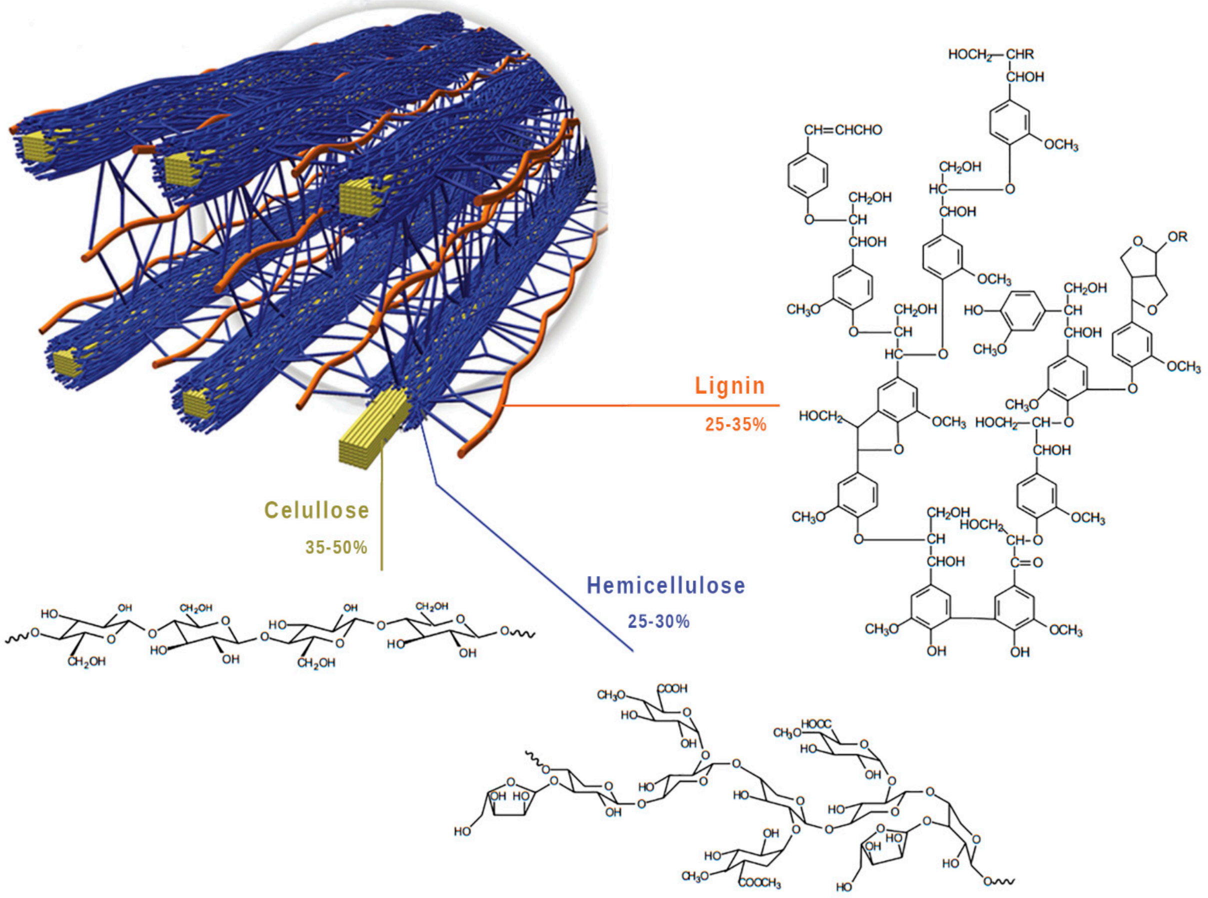

Figure S1. Spatial and chemical structure of the major polymeric constituents of wood: cellulose (yellow), hemicellulose (blue) and lignin (red). Represented chemical structure of lignin as well as content range are typical of a softwood. Image modified from references [15] and [16].

Table S1. Comparison of literature about characterization and biodegradation in compost/soil of PHA/lignocellulose composites.

Ref.	Matrix	Filler	Thickness	Material characterization	Biodegradability			Microbiological studies
					Disintegration	Mineralization	Environment	
[20]	PHBV	30-40% bamboo	2 mm (injected)	DMA, HDT, TGA, DSC, FTIR, SEM, MP*	--	--	--	--
[21]	PHBV	20% abaca fiber	2 mm (injected)	SEM, MP*, FTIR	**			
[22]	PHA	10 to 40% wood flour	1 mm	FTIR, XRD, DMA, DSC, MP*, SEM	--	--	--	Antibacterial assay
[23]	PHB	10-20 phr rice husk, seagrass, almond shell	350 µm	SEM, TGA, DSC, MP*, TF, barrier prop.	100% in 35 days	--	Compost 58°C	--
[24]	PHB	10-30-50% wood flour	Pellets & granules	FTIR, DSC, XRD, MP*, SEM	33-23% in 35 days	--	Soil	--
[25]	PHA	20-40% palm fiber	500 µm	FTIR, SEM, MP*.	60-90% in 60 days	--	Soil	Cell viability
[26]	PHBV	10-20-30% wheat straw fibers	1 mm	TGA, DSC, SEM, MP*	44-47% in 67 days // 20-23% in 6 months	55-65% in 48 days (in activated sludge)	Compost 50°C // Soil	--
[27]	PHBV	20-50% wood flour	1.6 mm	SEM; MP*	6.7-12.7% in 12 months	35-36% in 12 months	Soil	--
[28]	PHBV	Flax fibers	1 mm	SEM, TGA, FTIR	--	86% in 100 days	Compost 58°C	--
[29]	PHBV	20% seagrass	1.35 mm	SEM	9-23% in 12 months	--	*Marine (sediment)*	DNA sequencing
[30]	PHA	--	Microplastics	SEM, EDS, FTIR	--	--	Compost 50°C	DNA sequencing
This study	PHBV	15% woodflour	400 µm	SEM, rheology, TGA	100% in 45 days	97% in 60 days	Compost 58°C	Microbial count

*MP = Mechanical properties / **This reference states the composites studied are biodegradable. However, biodegradability was not checked.

Text S1. Calculation of the Polymer Molecular Weight via Intrinsic Viscosity Determined by Diluted Solutions Method

The molecular weight (*Mw*) of the PHBV composites at different composting times was calculated from intrinsic viscosity using the Mark-Houwink-Sakurada equation (Eq. S1):

$$M_w = K \cdot [\eta]^{\alpha} \tag{S1}$$

where [η] is the intrinsic viscosity and K and α are the constants for the specific polymer/solvent/temperature pair, being K = 1.25 x10^{-4} dL/g and α = 0.80 the values for PHBV/TFE [40]. Intrinsic viscosity, defined as the ratio of specific (η_{sp}) or relative viscosity (η_r) to concentration in the limit of infinite dilution (Eq. S2), was determined following a diluted solution procedure [41].

$$[\eta] = \lim_{c \to 0} \frac{\eta_{sp}}{c} = \lim_{c \to 0} \frac{\eta_r - 1}{c} \tag{S2}$$

PHBV and composites were dissolved in TFE at room temperature to prepare solutions of 5, 2.5, 1.25, and 0.625 g/L by serial dilution and filtered through a 0.2 µm membrane. Viscosity measurements were performed in a Cannon-Fenske viscometer at a controlled temperature of 30°C in a water bath. Flow times of the solution (t) and the pure solvent (t_0) were registered to determine the relative viscosity as the ratio between them (Eq. S3):

$$\eta_r = \eta/\eta_0 = t/t_0 \tag{S3}$$

Subsequently, [η] was calculated according to the Kraemer equation (Eq. S4), as the intercept in the linear least-squares fit of the inherent viscosity, η_{inh} = (ln η_r)/c, plotted against the concentration of the solutions (c) [42]:

$$\frac{\ln \eta_r}{c} = [\eta] - k_K[\eta]^2 c \tag{S4}$$

where k_K is the dimensionless Kraemer constant.

Text S2. Characterization of Fibers: WAXS, TGA and FTIR

Regarding crystalline structure, both fibers show a typical cellulose I XRD pattern (Fig. S2a). Three peaks can be observed at 2θ of approximately 16°, 22° and 34° which correspond to planes (110) and $(1\bar{1}0)$ merged in one peak, (200) and (004) respectively [45,46]. The presence of amorphous hemicellulose and lignin in WF broadens the (110) peak and reduces the intensity of (004), thus indicating an overall lower crystallinity.

The thermal decomposition of woodflour components overlaps between 200° and 500°C (Fig. S2b): hemicellulose decomposes first between 200°-340°C, cellulose degradation occurs from 300°to 400°C while lignin degrades slowly over the whole range resulting in a diffuse or not present peak [47]. The onset ($T_{5\%}$) of WF decomposition is 15°C lower than that of TC90 (see Table 1) which may be attributed to the higher hemicellulose content and moisture adsorbed by woodflour. However, when compared to TC90 cellulose, $T_{max.f}$ of WF (taken as that of the cellulose component) shifted from 351°C to 359°C. Wang et al. [48] reported that lignin enhances the thermal stability of cellulose due to the presence of stable aromatic units with a very low reactivity in an oxygen-deficient atmosphere [48,49].

FTIR spectra (see Fig. S2c) show that main bands of TC90 cellulose are also detected in WF spectrum since woodflour composition is a mixture of lignin, hemicellulose, and cellulose. The band at 896 cm^{-1}, is considered an IR crystallinity index [50,51], being its relative intensity a reference to the crystallinity of the cellulose. As expected, this band is more intense in TC90 than in WF spectrum. The spectrum of WF shows four extra peaks: 1730 cm^{-1} corresponds to C=O stretching band of acetyl groups in hemicellulose and carbonyl aldehyde in lignin [16], 1600 and 1508 cm^{-1} are ascribed to C=C stretching vibration of aromatic rings of lignin [16,52,53], and 1263 cm^{-1} is assigned to C-O stretch of guaiacyl ring structure of lignin [52,53].

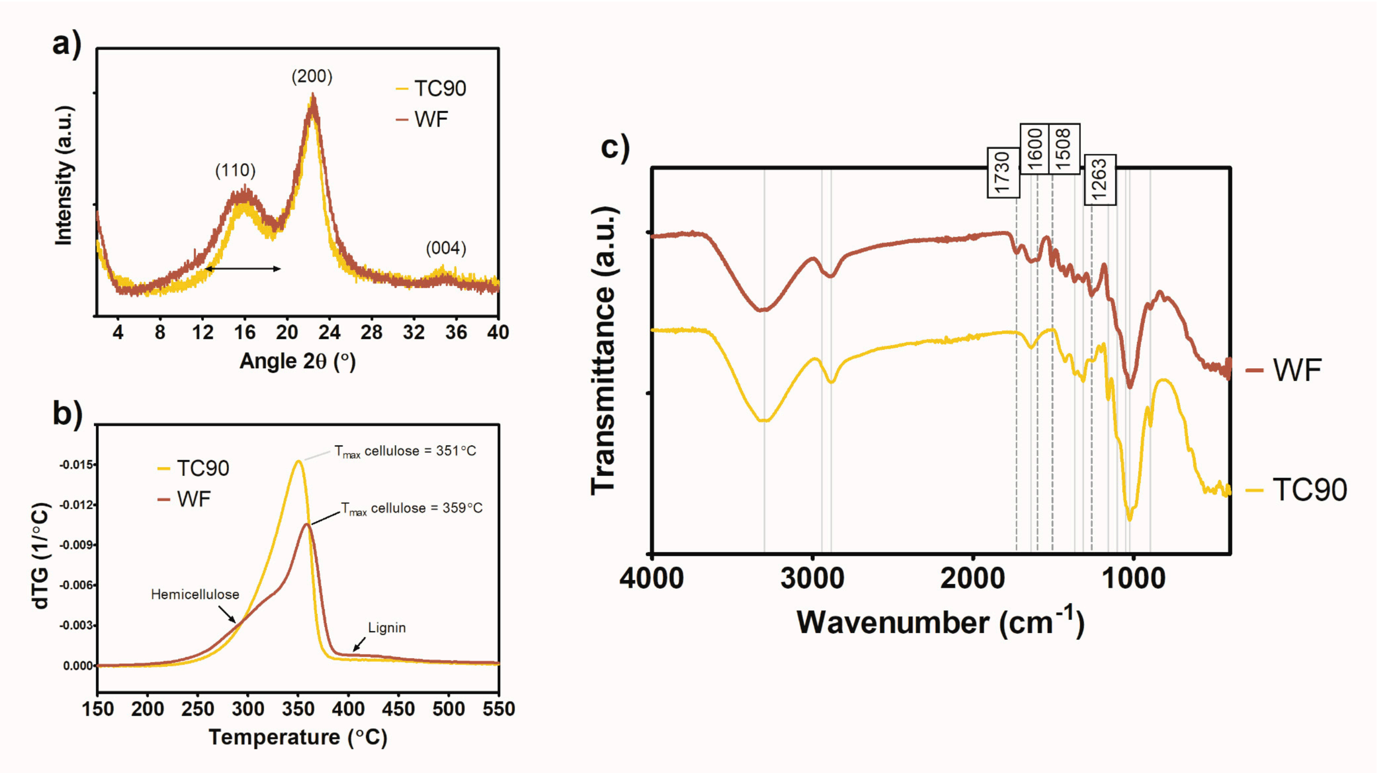

Figure S2. a) WAXS patterns of TC90 and WF. b) dTG curves of fibers. c) FTIR spectra of cellulose and woodflour. Diagnostic peaks of cellulose are marked by solid lines while diagnostic peaks of lignin are marked by dotted lines with wavenumbers above in a box.

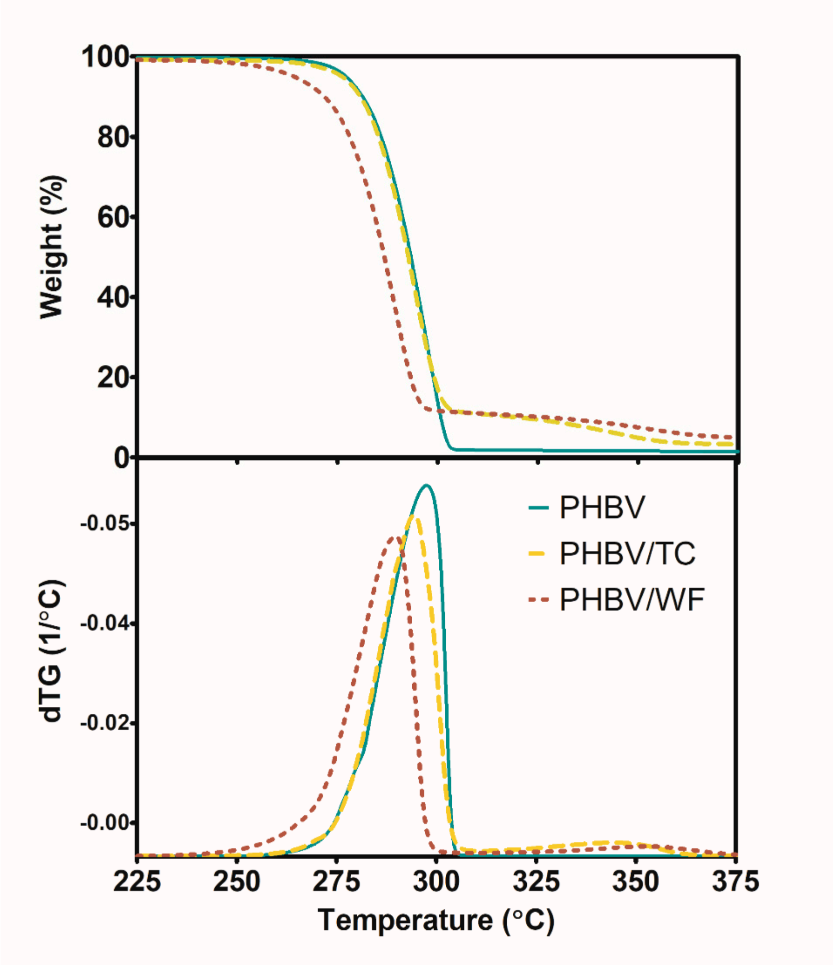

Figure S3. TG (up) and dTG (down) curves of PHBV-fiber composites.

References

15. Brandt, A.; Gräsvik, J.; Hallett, J.P.; Welton, T. Deconstruction of lignocellulosic biomass with ionic liquids. *Green Chem.* **2013**, *15*, 550–583. https://doi.org/10.1039/C2GC36364J
16. Barker, B.; Owen, N.L. Identifying softwoods and hardwoods by infrared spectroscopy. *J. Chem. Edu.* **1999**, *76*, 1706. https://doi.org/10.1021/ed076p1706
20. Singh, S.; Mohanty, A.K.; Sugie, T.; Takai, Y.; Hamada, H. Renewable resource based biocomposites from natural fiber and polyhydroxybutyrate-co-valerate (PHBV) bioplastic. *Compos. -A: Appl. Sci. Manuf.* **2008**, *39*, 875–886. https://doi.org/10.1016/j.compositesa.2008.01.004
21. Shibata, M.; Takachiyo, K.I.; Ozawa, K.; Yosomiya, R.; Takeishi, H. Biodegradable polyester composites reinforced with short abaca fiber. *J. Appl. Polym. Sci.* **2002**, *85*, 129–138. https://doi.org/10.1002/app.10665
22. Wu, C.S.; Liao, H.T. Fabrication, characterization, and application of polyester/wood flour composites. *J. Polym. Eng.* **2017**, *37*, 689–698. https://doi.org/10.1515/polyeng-2016-0284
23. Sánchez-Safont, E.L.; Aldureid, A.; Lagarón, J.M.; Gámez-Pérez, J.; Cabedo, L. Biocomposites of different lignocellulosic wastes for sustainable food packaging applications. *Compos. B. Eng.* **2018**, *145*, 215–225. https://doi.org/10.1016/j.compositesb.2018.03.037
24. Thomas, S.; Shumilova, A.; Kiselev, E.; Baranovsky, S.; Vasiliev, A.; Nemtsev, I.; Kuzmin, A.P.; Sukovatyi, A.; Avinash, R.P.; Volova, T. Thermal, mechanical and biodegradation studies of biofiller based poly-3-hydroxybutyrate biocomposites. *Int. J. Biol. Macromol.* **2020**, *155*, 1373–1384. https://doi.org/10.1016/j.ijbiomac.2019.11.112
25. Wu, C.S.; Liao, H.T.; Cai, Y.X. Characterisation, biodegradability and application of palm fibre-reinforced polyhydroxyalkanoate composites. *Polym. Degrad. Stab.* **2017**, *140*, 55–63. https://doi.org/10.1016/j.polymdegradstab.2017.04.016
26. Avella, M.; La Rota, G.; Martuscelli, E.; Raimo, M.; Sadocco, P.; Elegir, G.; Riva, R. Poly(3-hydroxybutyrate-co-3-hydroxyvalerate) and wheat straw fibre composites: Thermal, mechanical properties and biodegradation behaviour. *J. Mater. Sci.* **2000**, *35*, 829–836. https://doi.org/10.1023/A:1004773603516
27. Chan, C.M.; Vandi, L.J.; Pratt, S.; Halley, P.; Richardson, D.; Werker, A.; Laycock, B. Insights into the biodegradation of PHA/wood composites: Micro- and macroscopic changes. *Sustain. Mater. Technol.* **2019**, *21*, e00099. https://doi.org/10.1016/j.susmat.2019.e00099

28. Muniyasamy, S.; Ofosu, O.; Thulasinathan, B.; Thondi Rajan, A.S.; Ramu, S.M.; Soorangkattan, S.;Muthuramalingam, J.B.; Alagarsamy, A. Thermal- chemical and biodegradation behaviour of alginic acid treated flax fibers/poly (hydroxybutyrate-co-valerate) PHBV green composites in compost medium. *Biocatal Agric Biotechnol* **2019**, *22*, 101394. https://doi.org/10.1016/j.bcab.2019.101394
29. Vannini, C.; Rossi, A.; Vallerini, F.; Menicagli, V.; Seggiani, M.; Cinelli, P.; Lardicci, C.; Balestri, E. Microbial communities of polyhydroxyalkanoate (PHA)-based biodegradable composites plastisphere and of surrounding environmental matrix: a comparison between marine (seabed) and coastal sediments (dune sand) over a long-time scale. *Sci. Total Environ.* **2021**, *764*, 142814. https://doi.org/10.1016/j.scitotenv.2020.142814
30. Sun, Y.; Ren, X.; Rene, E.R.; Wang, Z.; Zhou, L.; Zhang, Z.; Wang, Q. The degradation performance of different microplastics and their effect on microbial community during composting process. *Bioresour. Technol.* **2021**, *332*, 125133. https://doi.org/10.1016/j.biortech.2021.125133
40. Bassett, D. *Developments in Crystalline Polymers—2*; Springer: Netherlands, 2012.
41. Pamies, R.; Hernández Cifre, J.G.; López Martínez, M.C.; García de la Torre, J. Determination of intrinsic viscosities of macromolecules and nanoparticles. Comparison of single-point and dilution procedures. *Colloid Polym. Sci.* **2008**, *286*, 1223–1231. https://doi.org/10.1007/s00396-008-1902-2
42. López Martínez, M.C.; Díaz Baños, F.G.; Ortega Retuerta, A.; García de la Torre, J. Multiple linear least-squares fits with a common intercept: Determination of the intrinsic viscosity of macromolecules in solution. *J. Chem. Edu.* **2003**, *80*, 1036. https://doi.org/10.1021/ed080p1036
45. French, A.D. Idealized powder diffraction patterns for cellulose polymorphs. *Cellulose.* **2014**, *21*, 885–896. https://doi.org/10.1007/s10570-013-0030-4
46. Miao, X.; Lin, J.; Bian, F. Utilization of discarded crop straw to produce cellulose nanofibrils and their assemblies. *J. Bioresour. Bioprod.* **2020**, *5*, 26–36. https://doi.org/10.1016/j.jobab.2020.03.003
47. Tranvan, L.; Legrand, V.; Jacquemin, F. Thermal decomposition kinetics of balsa wood: Kinetics and degradation mechanisms comparison between dry and moisturized materials. *Polym. Degrad. Stab.* **2014**, *110*, 208–215. https://doi.org/10.1016/j.polymdegradstab.2014.09.004
48. Wang, Y.; Liu, S.; Wang, Q.; Ji, X.; Yang, G.; Chen, J.; Fatehi, P. Strong, ductile and biodegradable polylactic acid/lignin-containing cellulose nanofibril composites with improved thermal and barrier properties. *Ind. Crops Prod.* **2021**, *171*, 113898. https://doi.org/10.1016/j.indcrop.2021.113898
49. Vanska, E.; Vihela, T.; Peresin, M.S.; Vartiainen, J.; Hummel, M.; Vuorinen, T. Residual lignin inhibits thermal degradation of cellulosic fiber sheets. *Cellulose.* **2016**, *23*, 199–212. https://doi.org/10.1007/s10570-015-0791-z
50. Abidi, N.; Cabrales, L.; Haigler, C.H. Changes in the cell wall and cellulose content of developing cotton fibers investigated by FTIR spectroscopy. *Carbohydr. Polym.* **2014**, *100*, 9–16. https://doi.org/10.1016/j.carbpol.2013.01.074
51. Xiong, J.; Li, Q.; Shi, Z.; Ye, J. Interactions between wheat starch and cellulose derivatives in short-term retrogradation: Rheology and FTIR study. *Food Res. Int.* **2017**, *100*, 858–863. https://doi.org/10.1016/j.foodres.2017.07.061
52. Colom, X.; Carrillo, F. Comparative study of wood samples of the northern area of Catalonia by FTIR. J. *Wood Chem. Technol.* **2005**, *25*, 1–11. https://doi.org/10.1081/WCT-200058231
53. Gelbrich, J.; Mai, C.; Militz, H. Evaluation of bacterial wood degradation by Fourier Transform Infrared (FTIR) measurements. *J. Cult. Herit.* **2012**, *13*(3), S135–S138. https://doi.org/10.1016/j.culher.2012.03.003
